# The Function of Rho-Associated Kinases ROCK1 and ROCK2 in the Pathogenesis of Cardiovascular Disease

**DOI:** 10.3389/fphar.2015.00276

**Published:** 2015-11-20

**Authors:** Svenja Hartmann, Anne J. Ridley, Susanne Lutz

**Affiliations:** ^1^Institute of Pharmacology, University Medical Center Göttingen, Georg-August-University Göttingen, Göttingen, Germany; ^2^German Center for Cardiovascular Research, Göttingen, Germany; ^3^Randall Division of Cell and Molecular Biophysics, King’s College London, London, UK

**Keywords:** Rho-kinase, ROCK, ROCK signaling, cardiovascular disease, heart, inhibitor, hypertrophy, fibrosis

## Abstract

Rho-associated kinases ROCK1 and ROCK2 are serine/threonine kinases that are downstream targets of the small GTPases RhoA, RhoB, and RhoC. ROCKs are involved in diverse cellular activities including actin cytoskeleton organization, cell adhesion and motility, proliferation and apoptosis, remodeling of the extracellular matrix and smooth muscle cell contraction. The role of ROCK1 and ROCK2 has long been considered to be similar; however, it is now clear that they do not always have the same functions. Moreover, depending on their subcellular localization, activation, and other environmental factors, ROCK signaling can have different effects on cellular function. With respect to the heart, findings in isoform-specific knockout mice argue for a role of ROCK1 and ROCK2 in the pathogenesis of cardiac fibrosis and cardiac hypertrophy, respectively. Increased ROCK activity could play a pivotal role in processes leading to cardiovascular diseases such as hypertension, pulmonary hypertension, angina pectoris, vasospastic angina, heart failure, and stroke, and thus ROCK activity is a potential new biomarker for heart disease. Pharmacological ROCK inhibition reduces the enhanced ROCK activity in patients, accompanied with a measurable improvement in medical condition. In this review, we focus on recent findings regarding ROCK signaling in the pathogenesis of cardiovascular disease, with a special focus on differences between ROCK1 and ROCK2 function.

## Similarities and Differences of ROCK1 and ROCK2 in Structure and Expression

**FIGURE 1:**
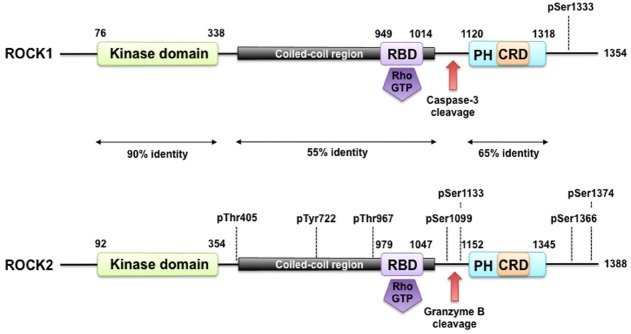
**ROCK structure and modes of regulation.** ROCK1 and ROCK2 consist of an N-terminally located kinase domain and a C-terminally located pleckstrin homology (PH) domain containing a cysteine-rich C1 domain (CRD). The region between the ROCK kinase domain and the PH domain forms a coiled coil structure, in which the Rho binding domain (RBD) is located. Both are highly homologous and share overall 64% amino acid sequence identity. A splice variant of ROCK2 contains an insertion of 57 amino acids following the RBD and is called ROCK2m. ROCK1 and ROCK2 can be activated by binding of RhoGTP to the RBD and through cleavage of ROCK1 by caspase-3 and of ROCK2 by granzyme B and caspase-2. Autophosphorylation of ROCK1 at Ser1333 and of ROCK2 at Ser1366 reflects the activation status of the kinases. Phosphorylation of ROCK2 at Thr967, Ser1099, Ser1133, or Ser1374 increased its activation status, whereas phosphorylation of Tyr722 decreases the ability of ROCK2 to bind to RhoA. Interaction of Thr405 of ROCK2 with the N-terminal extension of the ROCK2’s kinase domain is essential for substrate phosphorylation and kinase domain dimerization.

The Rho-associated kinases ROCK1 and ROCK2 belong to the family of serine/threonine AGC kinases named after the best investigated family members protein kinase A (PKA), G, and C. The structure of ROCK1 and ROCK2, including important regulatory phosphorylation and cleavage sites can be seen in Figure 1. ROCK1 and ROCK2 share overall 65% identity in their amino acid sequences (Nakagawa et al., 1996). ROCK1 and ROCK2 consist of 1354 and 1388 amino acids, respectively, and both contain an N-terminally located kinase domain, a coiled–coiled region followed by a Rho-binding domain (RBD; Ishizaki et al., 1996). The RBD binds exclusively to the switch I and switch II regions of GTP-bound active RhoA, RhoB, and RhoC (Shimizu et al., 2003; Dvorsky et al., 2004). At the C-terminus, there is a split PH-domain containing a cysteine-rich C1 domain (Wen et al., 2008). ROCK1 and ROCK2 have the highest amino acid homology between their kinase domain (92%), and are most divergent within their coiled-coil domains with 55% homology. Both full length kinases have similar kinase activity *in vitro* (Doran et al., 2004), consistent with the high homology of the kinase domains. No difference in affinity of the RBD of ROCK1 and ROCK2 for RhoA, RhoB, or RhoC has been described. The C-terminal PH-C1 tandem of ROCKs has been reported to play an autoinhibitory role by sequestering the N-terminal kinase domain and reducing its kinase activity (Wen et al., 2008). In addition, the PH-C1 tandem functions cooperatively in binding to membrane bilayers via the unconventional positively charged surfaces on each domain (Wen et al., 2008). However, the PH-C1 domains of ROCK1 and ROCK2 seem to have differential binding preferences for membrane lipids: the PH-C1 domain of ROCK2 was demonstrated to bind strongly to phosphatidylinositol (3,4,5)-trisphosphate and phosphatidylinositol (4,5)-bisphosphate, whereas the one of ROCK1 did not (Yoneda et al., 2005).

This might partly explain why ROCK1 and ROCK2 were described to have distinct subcellular distributions, which vary, however, depending on the cell type and method. For ROCK2 a cytosolic and nuclear localization, association with the centrosome, and co-localization with actin and vimentin filaments in different cell types were reported, as well as its localization at the intercalated disk and Z-disk of striated muscle cells (Leung et al., 1995; Matsui et al., 1996; Sin et al., 1998; Katoh et al., 2001; Kawabata et al., 2004; Ma et al., 2006; Tanaka et al., 2006; Iizuka et al., 2012). Further studies point to a role for ROCK2 in the formation of the contractile ring during cytokinesis, as it accumulates within the cleavage furrow during late mitosis (Kosako et al., 1999). In contrast to ROCK2, there is less information on the subcellular localization of ROCK1. However, it has been reported to have a cytosolic localization, as well as association with centrosomes, the plasma membrane, cell–cell contacts, cell adhesion sites, and vesicles (Chevrier et al., 2002; Glyn et al., 2003; Stroeken et al., 2006; Iizuka et al., 2012).

In addition to potential differences in subcellular localization, the expression of ROCK1 and ROCK2 varies between different tissues. In mouse, ROCK1 mRNA is ubiquitously expressed except in the brain and muscle, whereas ROCK2 mRNA is expressed abundantly in the brain, muscle, heart, lung, and placenta (Nakagawa et al., 1996). In a recent review, Julian and Olson (2014) analyzed the expression of both kinases based on expressed sequence tags, confirming the higher abundance of ROCK2 in heart and brain and suggesting a more prominent expression of ROCK1 in blood cells and the thymus.

In human and mouse skeletal muscle a unique splice variant of ROCK2 was detected, named ROCK2m. This variant contains an insertion of 57 amino acids following the RBD, and is progressively expressed during myogenic differentiation together with ROCK2. Low levels of ROCK2m expression were also consistently detected in heart and skin. Whether ROCK2m is differentially regulated, or has a different function to ROCK2 is not clear so far (Pelosi et al., 2007).

## Similarities and Differences in the Regulation of ROCK1 and ROCK2

### General Mechanism of ROCK Activation

Both the N-terminal and the C-terminal regions of ROCKs affect catalytic activity (Leung et al., 1996; Jacobs et al., 2006; Yamaguchi et al., 2006) and both ROCKs form head-to-head homodimers (Jacobs et al., 2006; Yamaguchi et al., 2006). This dimerization is dependent on an N-terminal extension region of the kinase domains, the kinase domain itself, as well as the coiled-coil domain. The active sites of the two kinases are thereby positioned in the same direction, possibly facilitating interactions with dimeric substrates. The C-terminal region of ROCKs, including the split PH domains, acts as an autoinhibitory domain by binding directly to the kinase interface: deletion of this part leads to a constitutive activation *in vitro* and *in vivo* (Leung et al., 1996; Ishizaki et al., 1997; Amano et al., 1999; Jacobs et al., 2006; Yamaguchi et al., 2006). Upon interaction of the RBD domain with GTP-loaded RhoA, ROCK activity increases through a mechanism called derepression: the repression of the kinase domain by the C-terminal region is removed, which subsequently leads to an active “open” conformation of the kinase domain. Whether this simple mechanism is supported or further fine-tuned by (auto)phosphorylation is not fully understood so far.

Similar to other members of the AGC kinase family, ROCK1 and ROCK2 contain a hydrophobic motif near the C-terminus of the kinase domain. Biochemical and structural studies of other AGC kinases have shown that this motif, especially when phosphorylated, promotes the active conformation of these kinases. ROCKs share a similar hydrophobic motif and for ROCK2 it has been shown that the threonine at position 405 in the hydrophobic motif is essential for substrate phosphorylation and kinase domain dimerization. However, in contrast to other kinases like Akt, this does not involve phosphorylation of Thr405 but interaction with the N-terminal extension of the kinase domain. In addition, the crystal structures of the ROCK catalytic domains indicate that, unlike many other protein kinases, phosphorylation of the activation loop of ROCK is not required for full catalytic activity (Jacobs et al., 2006; Yamaguchi et al., 2006). However, Polo-like kinase-1 was shown to phosphorylate ROCK2 at Thr-967, Ser-1099, Ser-1133, or Ser-1374, thereby acting together with RhoA to activate ROCK2 (Lowery et al., 2007). In contrast, phosphorylation of Tyr-722 decreases the ability of ROCK2 to bind to RhoA (Lee et al., 2010), whereas dephosphorylation increases the binding (Lee and Chang, 2008). Although it appears that ROCK activity is not dependent on (auto)phosphorylation, several phospho-sites in both ROCKs have been described. Of special experimental interest might be the autophosphorylation of ROCK1 at Ser1333 and of ROCK2 at Ser1366, as both phosphorylations were suggested to reflect the activation status of these kinases (Chuang et al., 2012, 2013). Isoform-specific antibodies against both phosphosites, as described by Chuang et al. (2012, 2013), provide a new tool to study ROCK activation, which is normally monitored indirectly through changes in myosin light chain (MLC) phosphorylation.

### Specific Mechanisms of ROCK1 Regulation

In addition to being activated by Rho subfamily GTPases and phosphorylation, ROCKs can be activated in other ways that are isoform-specific.

An early event in apoptosis is activation of caspases, leading to cleavage of many proteins, including ROCK1. ROCK1 is cleaved by caspase-3 at a conserved DETD1113/G sequence, thereby removing the autoinhibitory C-terminal region and thus resulting in constitutively active ROCK1 (Sebbagh et al., 2001). This leads to actomyosin-dependent membrane blebbing (Sebbagh et al., 2001), which contain fragmented DNA (Coleman et al., 2001). In heart tissues from patients with heart failure the cleaved ROCK1 fragment, as well as active caspase-3 was detectable, but was almost absent in normal hearts and in an equivalent cohort of patients with left ventricular assist devices (Chang et al., 2006). In animal and cell based models, downregulation of ROCK1 decreased cardiomyocyte apoptosis and interestingly caspase-3 activation. This could be due to an Akt-dependent positive feedback loop linking active ROCK1 to caspase-3 activation, resulting in a further augmentation of apoptosis (Chang et al., 2006).

The detrimental role of cleaved ROCK1 for the heart was further confirmed by transgenic overexpression of C-terminally truncated ROCK1 (ROCK1Δ1) in cardiomyocytes (Yang et al., 2012). These mice showed an increase in cardiac fibrosis, which was further augmented by angiotensin II treatment resulting in a fibrotic cardiomyopathy with diastolic dysfunction. ROCK1-mediated activation of the transcription factor, serum response factor, led to upregulation of TGFβ1 and hence activation of adjacent cardiac fibroblasts, which could explain the observed fibrosis. However, it is likely that ROCK1-induced cardiomyocyte death also contributes to cardiac fibrosis (Yang et al., 2012).

Initially, Rnd3 (also called RhoE) was thought to directly bind to ROCK1, thus inhibiting downstream signaling. However, recent data suggests that the Rnd3-dependent inhibition is indirect and mediated by a Rnd3-p190RhoGAP-dependent inhibition of Rho/ROCK signaling. Rnd3 belongs to the Rnd subfamily, which are atypical Rho family proteins. In contrast to other Rho GTPases, Rnd proteins can bind GTP, however, they are not able to hydrolyze it and are therefore not regulated by the classical GTP/GDP conformational switch of small GTPases (Riou et al., 2010). Rnd3 activity itself is inhibited by ROCK1-dependent phosphorylation on multiple sites, which induces its translocation from the membrane to the cytosol and reduces its ability to induce loss of stress fibers (Riento et al., 2003, 2005; Riou et al., 2013).

With respect to the heart, Rnd3 haploinsufficient mice are predisposed to left ventricular pressure overload and develop severe heart failure after transverse aortic constriction (TAC; Yue et al., 2014). Intriguingly, following TAC operation the cardiomyocyte apoptosis rate was higher compared to control animals, correlating with an increase in cleaved caspase-3. In addition, ROCK activity was increased. Homozygous Rnd3-null mice died *in utero* at embryonic stage E11 and isolated hearts from E10.5 showed a severe apoptotic cardiomyopathy along with elevated ROCK activity. By crossbreeding the haploinsufficient Rnd3 with the global ROCK1-knockout (KO) mice, the augmented effects observed after TAC operation were strongly reduced, thus arguing for a role of Rnd3-ROCK1 signaling in the regulation of cardiomyocyte apoptosis in heart disease (Yue et al., 2014). Recently, the function of Rnd3 in the heart was broadened by demonstrating that β2-adrenergic receptor density is increased in embryonic hearts from Rnd3-null mice due to an impaired receptor internalization and degradation in lysosomes. As a consequence, Rnd3-null mice died between E10.5 and E12.5 with fetal arrhythmias, probably due to β2-adrenergic receptor dependent PKA hyperactivity resulting in severe Ca^2+^ leakage through destabilized ryanodine receptor type 2 Ca^2+^ release channels (Yang et al., 2015). Whether this involves Rnd3-dependent Rho/ROCK inhibition or is dependent on a different Rnd3-regulated mechanism is not known.

The interaction of ROCK1 and Rnd3 is counter-regulated by the phosphoinositide-dependent kinase-1 (PDK1), which competes directly with Rnd3 for binding to ROCK1. In the absence of PDK1, negative regulation of Rho/ROCK signaling by Rnd3 predominates, causing reduced actomyosin contractility and motility as shown for cancer cells. The binding of PDK1 to ROCK1 was independent of protein phosphorylation (Pinner and Sahai, 2008). So far, regulation of ROCK by PDK1 has not been investigated in the cardiovascular system.

### Specific Mechanisms of ROCK2 Regulation

Like ROCK1, ROCK2 is cleaved and activated by proteases. Natural killer cells and cytotoxic T-cells can induce apoptosis in target cells by two distinct mechanisms: a caspase-dependent mechanism and by exocytosis of cytotoxic granules mainly releasing perforin and the protease granzyme B (Barry and Bleackley, 2002). Sebbagh and colleagues demonstrate that during incubation of lymphokine-activated killer cells together with myelogenous leukemia K562 target cells a caspase-independent cleavage of ROCK2 occurred in K562 cells. ROCK2 was demonstrated to be cleaved by granzyme B at the IGLD^1131^ sequence, which is absent in ROCK1. This cleavage at the C-terminus resulted in a constitutively active ROCK2 fragment of 130 kDa in size. Subsequently to ROCK2 cleavage, caspase activation occurred and thus cleavage and activation of ROCK1 (Sebbagh et al., 2005). Additionally, granzyme B cleaves components of the extracellular matrix, thereby leading to the release of growth factors like TGFβ1 (Boivin et al., 2012) and to vascular wall instability and aneurysm (Chamberlain et al., 2010). A role for granzyme B in diverse cardiovascular diseases including myocarditis and atherosclerosis has been discussed (Saito et al., 2011), however, no link to ROCK2 has so far been established within this context.

As well as granzyme B, caspase-2 cleaves and activates ROCK2. Upon thrombin stimulation ROCK2, but not ROCK1, was upregulated and cleaved by caspase-2 into a 140 kDa fragment in endothelial cells. This led to an increase in the release of microparticles (Sapet et al., 2006). The specificity of caspase-2 for ROCK2 was later confirmed in endothelial cells treated with the Na^+^/K^+^-ATPase inhibitor ouabain. In these experiments an additional ouabain-induced cleavage of ROCK1 by caspase 3 was described altogether leading to apoptosis (Ark et al., 2010). Ouabain is used experimentally to study effects of cardiac glycosides like digoxin but more interestingly was shown to be an endogenous cardiotonic steroid upregulated in essential hypertension, heart and kidney failure (Blaustein, 2014).

As mentioned above, the PH domain of ROCK2 binds to phosphatidylinositol (3,4,5)-trisphosphate (PIP_3_) and phosphatidylinositol (4,5)-bisphosphate (PIP_2_), whereas the PH domain of ROCK1 does not (Yoneda et al., 2005). In contrast, in ROCK1-null bone marrow macrophages an increased phosphatase and tensin homolog deleted on chromosome 10 (PTEN) cleavage, reduced phosphorylation, stability, and activity has been demonstrated, which was accompanied by an enhanced activation of downstream PTEN targets, including AKT, GSK-3β, and cyclin D1 (Vemula et al., 2010). PTEN dephosphorylates PIP_3_ and thus counteracts PI3-kinase, which produces PIP_3_ (Oudit et al., 2004). Interestingly, it has been shown in neutrophils that PI3K activity is localized at the leading edge of the cells during chemotaxis, whereas PTEN is localized at the back (Li et al., 2005). This process of differential localization of PTEN and PI3K activity in the same cell contributes to restricted PIP_3_ levels in specific compartments like the leading edge during cell migration (Funamoto et al., 2002). Whether this interplay involves a differential activation of ROCK1 and ROCK2 has to be elucidated in the future.

Moreover, ROCK2 expression and activity were found to be modulated by the peripheral clock gene BMAL. Deletion of BMAL1 from smooth muscle in mice interfered with blood pressure circadian rhythm and decreased blood pressure, without affecting the central pacemaker suprachiasmatic nucleus (SCN). In mesenteric arteries, BMAL1 deletion suppressed the time-of-day-variations in response to agonist-induced vasoconstriction, associated with decreased myosin phosphorylation. BMAL1 was found to bind directly to the promoter of ROCK2 in a time-of-day-dependent manner, thereby regulating the time-of-day-variation in ROCK2 activity. However, it is not clear from the study whether ROCK1 might also be a BMAL1 target gene (Xie et al., 2015).

## ROCK Targets and the Cardiovascular System

**FIGURE 2:**
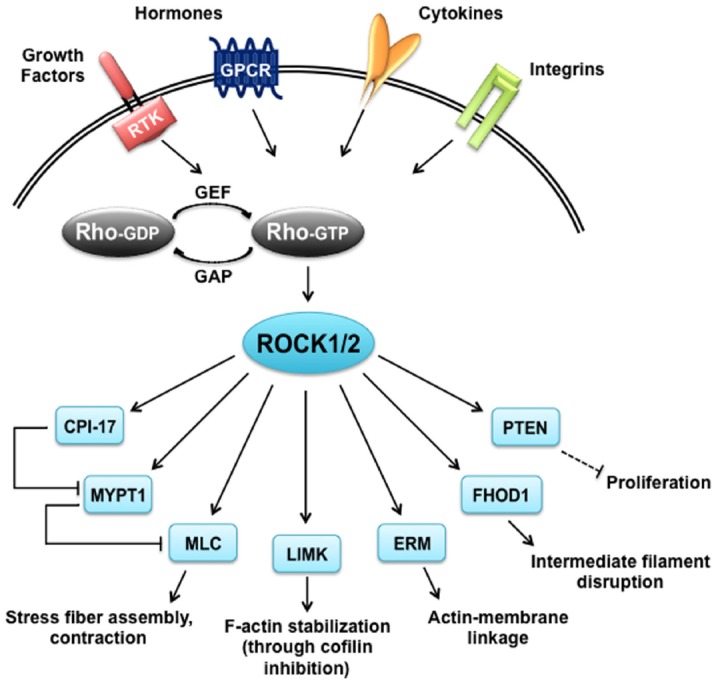
**ROCK targets.** Rho proteins can be activated by guanine nucleotide exchange factors (GEFs), which are themselves activated by receptor tyrosine kinases (RTKs), G protein coupled receptors (GPCRs), cytokines and integrins. Rho-GTP subsequently activates ROCK1 and ROCK2 that have a broad range of substrates and are responsible for diverse cellular responses. CIP-17, kinase C-potentiated phosphatase inhibitor of 17 kDa; ERM, ezrin-radixin-moesin; FHOD1, formin homology 2 domain-containing 1; GAP, GTPase activating protein; LIMK, LIM-kinase; MLC, myosin II regulatory light chain; MYPT1, myosin phosphatase target subunit 1; PTEN, phosphatase and tensin homolog deleted on chromosome 10.

Similar to many AGC kinases, both ROCK1 and ROCK2 phosphorylate the consensus sequence Arg/Lys–X–Ser/Thr or Arg/Lys–X–X–Ser/Thr, but other non-consensus sites have been identified as well (Kang et al., 2007; Riou et al., 2013). Although ROCKs share this consensus sequence with other kinases like PKA and PKG, the target overlap is supposedly minor based on the opposing functions of ROCKs and the cyclic nucleotide-regulated kinases in many tissues. In contrast, ROCKs seem to share substrates with PKCs, however, in some cases with different kinetics as reviewed below. The specificity of ROCK substrates has not been thoroughly analyzed for differences, which makes it difficult to draw a distinction between the two isoforms. To date, the only well-characterized specific substrate is Rnd3, which was shown to be phosphorylated by ROCK1 but not ROCK2 (Riento et al., 2005). An overview of vascular and cardiac ROCK substrates described in this review can be seen in Figure 2.

### Vascular ROCK Substrates

The best characterized ROCK substrate is the myosin phosphatase target subunit 1 (MYPT1), a regulatory subunit of protein phosphatase 1 (PP1), also called myosin-binding subunit (MBS) of myosin phosphatase. MYPT1 in complex with PP1 and the small subunit M20 counteracts the activity of MLC kinase and thus decreases the contraction of smooth muscle cells by dephosphorylating MLC. Depending on the species, ROCK phosphorylates MYPT1 at Thr-697, Ser-854 or Thr-855, which attenuates MLCP activity (Feng et al., 1999; Kawano et al., 1999) and induces the dissociation of MLCP from myosin II (Velasco et al., 2002). Thus, ROCK increases net MLC phosphorylation leading to actomyosin contraction.

In addition, ROCK phosphorylates protein kinase C-potentiated phosphatase inhibitor of 17 kDa (CPI-17) at Thr38 (Koyama et al., 2000). Initially, PKC was shown to phosphorylate CPI-17; however, it can be phosphorylated by several other kinases, including ROCK. This phosphorylation increases the phosphatase inhibitory potency of CPI-17 by over 1,000-fold (reviewed by Eto, 2009). Coordinated phosphorylation of CPI-17 by different kinases is believed to be important for regulating smooth muscle contraction. For example, α_1_-adrenergic receptor stimulation results in a biphasic phosphorylation of CPI-17 through sequential activation of PKC and ROCK. Following blockade of Ca^2+^ release from the sarcoplasmic reticulum by the ryanodine receptor, the α_1_-adrenergic receptor-induced, delayed and sustained activation of ROCK maintains CPI-17 and MYPT1 phosphorylation, causing tonic smooth muscle contraction (Dimopoulos et al., 2007). Moreover, the ROCK-dependent regulation of CPI-17 was further substantiated by analysis of adult MYPT1 smooth muscle-specific KO mice. It was shown that the observed enhanced phosphorylation of MLC and contractile force in isolated mesenteric arteries in response to vasoconstrictors could be inhibited by ROCK and PKC inhibitors, both resulting in diminished phosphorylation of CPI-17 (Qiao et al., 2014). There is also evidence that ROCK-dependent CPI-17 regulation plays a role in disease. The vascular smooth muscle contractile hyperactivity detected in isolated mesenteric arteries from type 2 diabetes db/db-mice was due to increased RhoA/ROCK activity and CPI-17 phosphorylation. This activation could also be mimicked by high glucose in isolated aortic smooth muscle cells (Xie et al., 2006). Finally, in the context of smooth muscle regulation, ROCK has been reported to directly phosphorylate Ser19 of MLC, which is the same residue phosphorylated by MLCK (Amano et al., 1996; Kureishi et al., 1997).

ERM (ezrin, radixin, and moesin) proteins are suggested to be downstream targets of ROCK. ERM proteins are closely related to each other and are thought to function as general crosslinkers between the plasma membrane and actin filaments. The last 34 C-terminal amino acids have been demonstrated to interact with actin filaments (Pestonjamasp et al., 1995). ROCK was shown to phosphorylate all three proteins at a threonine in this region *in vitro*. This phosphorylation interfered with the intramolecular and/or intermolecular head-to-tail association of ERM proteins, which is an important regulatory mechanism of their function (Matsui et al., 1998). In endothelial cells, ERM proteins have been shown to coordinate the localization of leukocyte adhesion molecules in a docking structure and play a role in barrier function, which is partly dependent on ROCK activity (Barreiro et al., 2002; Guo et al., 2009; Zhang et al., 2014). In bovine pulmonary artery endothelial cells the adapter molecule Na^+^/H^+^ exchanger regulatory factor 2 (NHERF2) was identified to be essential for ROCK–ERM interaction and ERM phosphorylation (Boratko and Csortos, 2013). However, other work suggests that in endothelial cells PKCs are the major activator of proteins (Adyshev et al., 2011, 2013). Also in smooth muscle, a role for ROCK-dependent ERM phosphorylation was established. In isolated arteries, ERM proteins were shown to be phosphorylated in response to vasoconstrictors involving PKC and ROCK. Interestingly, it was demonstrated that both kinases showed different kinetics similar as was described for CPI-17 phosphorylation (Koyama et al., 2000). ERM phosphorylation in smooth muscle cells led to binding of moesin to EBP50, which is an adaptor molecule, thereby regulating smooth muscle cell contractility (Baeyens et al., 2010).

LIM-kinases (LIMK1 and LIMK2) are well-characterized ROCK substrates, which in turn phosphorylate cofilin. This phosphorylation inhibits the actin-depolymerizing and actin-severing function of cofilin, and thus leads to an increase in actin filaments (Maekawa et al., 1999; Bravo-Cordero et al., 2013). LIMK expression is ubiquitous (Foletta et al., 2004). So far, LIMKs and cofilins have been mainly linked to neurological disorders and cancer. In endothelial cells it was demonstrated that shear stress, VEGF and thrombin induce ROCK-dependent LIMK activation and subsequent cofilin phosphorylation leading to sterol regulatory element binding protein 2 activation, rearrangements of the actin cytoskeleton, and a decrease in cofilin oligomerization, respectively (Lin et al., 2003; Gong et al., 2004; Goyal et al., 2013).

### ROCK Substrates in the Heart

In contrast to vascular cells, less is known about the roles of ROCK activity in cardiac cells and most work has focused on MLC phosphorylation. In most studies on ROCK-dependent signaling, the phosphorylation of MYPT1 was detected as a surrogate for ROCK activity (Okamoto et al., 2013; Yue et al., 2014). In heart tissue, the ROCK targets MYPT1 and the 75% homologous striated muscle-isoform MYPT2 are both expressed. Electron microscopy indicates that MYPT2 is located at, or close to, the Z-line, the A band and mitochondria (Fujioka et al., 1998; Okamoto et al., 2006). Upon α-adrenergic receptor stimulation, phosphorylation of MYPT and MLC2 was increased in adult rat ventricular cardiomyocytes, cardiomyocyte Ca^2+^ sensitivity of tension was also augmented and all these effects could be blocked by ROCK inhibition with Y-27632. Furthermore, activation of α-adrenergic receptors led to a ROCK-dependent decrease in the PP1-myofilament association (Rajashree et al., 2005). This spatial redistribution of PP1 could lead to changes in the phosphorylation pattern of PP1 substrates at other compartments than the sarcomere. A well-documented substrate of PP1 in cardiomyocytes is phospholamban, the regulator of the sarcoplasmic reticulum calcium ATPase (SERCA; Nicolaou et al., 2009). Interestingly, in adult rat ventricular cardiomyocytes phenylephrine was indeed shown to significantly reduce phospholamban phosphorylation leading to a decline in relaxation velocity (Jeong et al., 2010). In human atrial tissue, phenylephrine induced a concentration-dependent, sustained positive inotropic effect accompanied by an increase in MLC2 phosphorylation. In addition, in some tissues a small increase in relaxation time could be observed. Disinhibition of MLC phosphatase by ROCK inhibition partially reduced these effects. In contrast, in ventricular human tissue no influence of ROCK on the inotropic effect was observed and the effect of phenylephrine on MLC2 phosphorylation was not consistent (Grimm et al., 2005). In a tachypacing dog heart model, ROCK contributed to the phenylephrine-dependent increase in myofibrillar Ca^2+^ sensitivity in failing, but not in healthy ventricular cardiomyocytes. This was accompanied by a higher expression of Gα_q_ and RhoA, and an augmented phosphorylation level of MLC2 in the failing myocardium (Suematsu et al., 2001). In this context, it is important to mention that the phosphorylation of MLC2 in the healthy contracting heart is in steady state. The balance is kept by low activities of the MLC kinase and phosphatase, and not influenced by β-adrenergic signaling. Phosphorylation disappears in non-contracting, isolated hearts and in isolated cardiomyocytes, but can be restored by pacing (Chang et al., 2015) and α-adrenergic signaling as described above. This steady state phosphorylation status of MLC2 in the heart is not equally distributed, as it was demonstrated that MLC2 phosphorylation occurs in a decreasing gradient from epi- to endocardium and from apex to mid-ventricle with fiber-to fiber variations. This gradient is thought to play a role in ventricular torsion (Davis et al., 2001; Sheikh et al., 2012).

Other ROCK targets have been sporadically shown to be regulated in the heart, like ERM proteins (Zhang et al., 2006). In adult ventricular cardiomyocytes, phosphorylated ERM is localized at basal state mainly at intercalated disk regions. However, upon intracellular acidification, phosphorylated ERM was additionally detectable along the lateral sarcolemma and in small intracellular dots, and its overall amount was increased. This increase in phosphorylation could be partly inhibited by ROCK inhibition (Darmellah et al., 2009). Another ROCK target in cardiomyocytes is FHOD3, a diaphanous related formin, which plays a role in actin filament polymerizations. ROCK1 interacts with and activates FHOD3 involving its phosphorylation (Iskratsch et al., 2013). Also, ROCK-dependent cofilin phosphorylation was shown in adult cardiomyocytes. After treatment of these cells with adiponectin, an important regulator of peripheral energy metabolism, cofilin phosphorylation increased in a RhoA/ROCK-dependent manner. This lead to an increased vesicular trafficking of lipoprotein lipase to the surface of cardiomyocytes, suggesting a role of ROCK in metabolism (Ganguly et al., 2011). The muscle specific isoform cofilin-2 was shown in a KO mouse model to be important for development, as newborn KO mice were significantly smaller and weaker compared to their wild type littermates and died by day eight. Moreover, by day seven the skeletal muscles showed severe sarcomeric disruptions, accompanied by filamentous actin accumulation. The onset of pathology in cofilin-2 KO mice correlated with the normal developmental loss of cofilin-1 expression within myofibers. Interestingly, no cardiac muscle degeneration could be observed in the cofilin-2 KO mice, and high expression of cofilin-1 was found within cardiac myofibers, suggesting that cofilin-1 may be able to compensate for cofilin-2 deficiency in cardiac muscle (Agrawal et al., 2012). The importance of cofilin-2 for the heart was demonstrated recently. Haploinsufficiency of cofilin-2 in cardiomyocytes resulted in a dilated phenotype with increased left ventricular volume, and decreased wall thickness and contractile function. In these mice, myofibrils showed an abnormal actin pattern, lack of proper orientation, and a poorly organized sarcomere. Aggregates of sarcomere proteins occur similar to those observed in idiopathic dilated cardiomyopathy and in rare nemaline myopathy (Subramanian et al., 2015).

## Genetic Animal Models of ROCK1 and ROCK2

### General Knockout of ROCK1 and ROCK2

The first ROCK KO mouse model described was the global KO of ROCK2 (Thumkeo et al., 2003). Around 90% of ROCK2-KO mice died *in utero* due to a perturbed embryo–placenta interaction. The surviving animals were significantly smaller after birth, but subsequently caught up in growth. Histological analyses of tissues from the surviving adult animals did not reveal any changes compared to wild type littermates (Thumkeo et al., 2003). In contrast, the global KO of ROCK1 was less severe than that of ROCK2. Loss of ROCK1 resulted in failure of eyelid closure and closure of the ventral body wall. Most ROCK1 KO mice died soon after birth as a result of cannibalization by the mother. Altered actin cytoskeletal organization with decreased MLC phosphorylation was identified as the underlying cause of these phenotypes (Shimizu et al., 2005). However, the phenotype of the global ROCK1 KO seems to be dependent on the genetic C57BL/6 background of the mice. On the FVB background the phenotype was much stronger: 60% of the mice died *in utero* before E9.5 (Zhang et al., 2006). Similarly, the ROCK2 KO embryo–placenta interaction phenotype leading to death *in utero* on a C57BL/6 × 129/SvJ background was no longer present when the mice were bred onto a CD-1 (C57BL/6 × Dba) background (Duffy et al., 2009). Interestingly, ROCK2 KO mice on a C57BL/6 background showed, in addition to the placental dysfunction, a failure of eyelid and ventral body wall closure similar to ROCK1 KO mice (Thumkeo et al., 2005). These findings clearly demonstrate the impact of the genetic background on the phenotype of ROCK KO mice.

### Vascular Models

ROCKs are implicated in regulating blood pressure by influencing MLC phosphorylation as reviewed above. However, analysis of mouse models has not provided conclusive evidence for a link between ROCKs and blood pressure. In global haploinsufficient ROCK1 and ROCK2 mouse models, no change in basal blood pressure was observed (Noma et al., 2008). Similarly, in haploinsufficient ROCK1^+/–^ mice there was no difference in angiotensin II induced increase in blood pressure compared to control mice (Rikitake et al., 2005b). There was also no difference detected in blood pressure of mice with a vascular smooth muscle cell (VSMC)-specific, haploinsufficient ROCK2^+/–^ KO under basal conditions or after 4 weeks in hypoxia. Moreover, in the same study transgenic mice with almost twofold overexpression of ROCK2 in smooth muscle cells did not show any changes in blood pressure under normoxic or hypoxic conditions (Shimizu et al., 2013). In contrast, in similar haploinsufficient ROCK1^+/–^ and ROCK2^+/–^ KO mouse lines a decrease in blood pressure under basal conditions and an attenuation of the diabetes-induced blood pressure increase was observed (Yao et al., 2013). So far, the blood pressure has not been determined in mice lacking any ROCK expression in VSMC.

ROCKs have been implicated in processes after vascular injury mainly by use of ROCK inhibitors (Shibata et al., 2001, 2003; Matsumoto et al., 2004). Ligation of the common carotid artery in mice leads to leukocyte recruitment, neointima formation, and narrowing of the lumen of the vessel (Schober and Weber, 2005). Similar changes were observed in haploinsufficient ROCK2^+/–^ mice. In contrast, in haploinsufficient ROCK1^+/–^ mice, neointima formation was less pronounced and VSMC proliferation and survival, as well as expression levels of proinflammatory adhesion molecules, were decreased. Moreover, there was reduced leukocyte infiltration. Reciprocal bone marrow transplantation in wild type and ROCK1 heterozygotes indicated that leukocytes were primarily responsible for the observed differences (Noma et al., 2008). This could reflect the important roles of Rho/ROCK signaling in leukocyte migration, which has been extensively reviewed elsewhere (Biro et al., 2014).

There are several lines of evidence that ROCKs play a role in the pathology of diabetes. ROCK1 KO mice exhibit insulin resistance and can have a significant increase in glucose-induced insulin secretion, leading to hyperinsulinemia. Impaired insulin signaling in skeletal muscle could be responsible for these changes (Lee et al., 2009). Moreover, it was shown that ROCK1 KO mice treated with streptozotocin to induce diabetes were protected against the development of albuminuria, which is a typical sign of kidney dysfunction in diabetes. This was associated with protection against kidney fibrosis (Zhou et al., 2011). Under basal conditions the blood pressure in haploinsufficient ROCK1 or ROCK2 mouse models was lower compared to control mice and streptozotocin-induced increase was absent (Yao et al., 2013). However, there was no impact on the basal and streptozotocin-induced blood glucose levels. In diabetes, high glucose levels lead to a decrease in nitric oxide (NO) synthesis in the vascular endothelium and hence an impairment of vascular dilatation (Tousoulis et al., 2013). Accordingly, in aortic rings from diabetic wild type mice, NO levels were reduced and acetylcholine-dependent dilatation was impaired. In contrast, aortic rings from haploinsufficient ROCK1^+/–^ and ROCK2^+/–^ mice produced higher levels of NO under basal conditions, and a less pronounced difference in dilatation. Interestingly, reduced ROCK1 expression was more effective in preventing diabetes-induced changes than ROCK2. Up-regulation of arginase was reduced in haploinsufficient diabetic ROCK1 and ROCK2 mice, which could explain the attenuation of diabetes-induced vascular endothelial dysfunction and improvement in vascular function during diabetes (Yao et al., 2013). Studies in macrophages and vascular endothelial cells have shown that arginase can compete with nitric oxide synthase (NOS) for L-arginine, thereby limiting NO production. In this context, increased arginase activity has been associated with vascular dysfunction in diabetes and atherosclerosis (Romero et al., 2008). Moreover, hyperglycemia was shown to induce RhoA/ROCK signaling in macrophages, thereby activating JNK and ERK signaling cascades. This subsequently led to activation of macrophages and thus eventually to the development of atherosclerosis (Cheng et al., 2015).

Finally, ROCKs are implicated in the pathogenesis of pulmonary arterial hypertension (PAH), which is rare but usually fatal. ROCK2 but not ROCK1 expression is enhanced in the media of pulmonary arteries and pulmonary arterial smooth muscle cells from patients with idiopathic PAH (Shimizu et al., 2013). Conditional ROCK2 KO in VSMC in mice prevented the chronic hypoxia-induced increase in right ventricular systolic pressure, as well as the pulmonary vascular remodeling. On the other hand, transgenic overexpression of ROCK2 in VSMC augmented both characteristics of PAH. These findings correlated with changes in mitogenic characteristics in lung tissue including ERK1/2 phosphorylation. Isolated smooth muscle cells from the mouse aorta as well as pulmonary arterial smooth muscle cells from patients showed ROCK2-dependent regulation of cell proliferation, survival and migration (Shimizu et al., 2013).

### Cardiac Models

So far, no cardiomyocyte or cardiac fibroblast-specific KO model is available for ROCK1, although data from genetically modified ROCK1 mice point to a role in cardiac fibrosis. Cardiac fibrosis is driven by a complex interlinked mechanism which involves on the one hand the transdifferentiation of cardiac fibroblasts, endothelial cells, and other cell types into myofibroblasts, and on the other hand the dysfunction and apoptosis of cardiomyocytes. As ROCK1 was demonstrated to propagate apoptosis in cardiomyocytes (Chang et al., 2006), but also to influence cardiac fibroblast behavior (Haudek et al., 2009), to date it is not possible to determine whether ROCK1 primarily functions in cardiomyocytes, cardiac fibroblasts, or both.

Mice with a global ROCK1 deletion that underwent TAC to induce cardiac hypertrophy had reduced perivascular and interstitial cardiac fibrosis at 3 weeks, but not at 1 week after the banding. Expression and secretion of fibrotic cytokines such as the pro-fibrotic TGFβ2 and connective tissue growth factor (CTGF), as well as of extracellular matrix proteins was reduced. Interestingly, ROCK1 deletion did not impair the development of cardiac hypertrophy. This was supported by findings in transgenic mice with cardiomyocyte-specific overexpression of Gα_q_, which mimics pressure-overload induced hypertrophy, and homozygous KO of ROCK1. In these mice, the deletion of ROCK1 led to a decreased expression of the fibrogenic factors TGFβ2 and CTGF to the same extent as in hypertrophic hearts of ROCK1 KO mice (Zhang et al., 2006). In another transgenic model of Gα_q_ overexpression, ROCK1 deletion attenuated left ventricular dilation, contractile dysfunction and cardiomyocyte apoptosis, but did not prevent the development of cardiac hypertrophy. Interestingly, KO of ROCK1 reduced the induction of hypertrophic markers, suggesting that ROCK1 might at least be able to modify cardiac hypertrophy (Shi et al., 2008). Consistent with this, ROCK1 deficiency might have beneficial effects in preventing the transition from cardiac hypertrophy to heart failure. Mice with cardiac overexpression of Gα_q_ develop lethal cardiomyopathy after pregnancy or at old ages. Interestingly, deletion of ROCK1 resulted in improved survival, attenuated left ventricular dilation and wall thinning, and decreased cardiomyocyte apoptosis. In addition, contractile function was preserved after pregnancy and in 12-month-old mice. Moreover, overexpression of ROCK1 in transgenic Gα_q_ mice was associated with increased cardiomyocyte apoptosis and the development of decompensated cardiomyopathy, even in mice that were not subjected to pregnancy stress (Shi et al., 2010).

A role for ROCK1 in cardiac hypertrophy and remodeling was also addressed using haploinsufficient ROCK1^+/–^ mice, which were treated either with angiotensin II or NG-nitro-L-arginine methyl ester (L-NAME) to inhibit NOS. Both treatments led to a comparable increase in systemic blood pressure, left ventricular wall thickness and mass, and hypertrophy in haploinsufficient ROCK1 and wild type mice. However, similar to ROCK1 KO mice, the hearts of the haploinsufficient ROCK1 mice showed less perivascular fibrosis, associated with decreased expression of TGFβ, CTGF, and collagen III. Likewise, haploinsufficient ROCK1 mice, which were subjected to TAC or myocardial infarction showed decreased perivascular fibrosis (Rikitake et al., 2005b). Transgenic mice expressing a truncated active form of ROCK1 in cardiomyocytes developed fibrotic cardiomyopathy and showed elevated NF-κB signaling, as well as SRF-driven increased TGFβ1 signaling. One reasonable explanation for the effects on cardiac fibrosis in the different genetic ROCK1 mouse models is a positive feed-forward regulatory loop of ROCK1 and caspase-3, as discussed above (Chang et al., 2006; Yang et al., 2012).

The role of ROCK1 was further studied in a model of ischemia/reperfusion cardiomyopathy (I/RC) by occlusions of the left anterior descending artery. ROCK1 KO mice were protected from the development of I/RC-mediated myocardial dysfunction and had reduced cardiac fibrosis. This was accompanied by a lower number of CD34/CD45-positive cells, which are thought to be blood–borne monocytic fibroblast precursors being attracted to the myocardium in I/RC (Haudek et al., 2006). Interestingly, fibroblast formation from human peripheral blood mononuclear cells was impaired in cells with lower ROCK1 expression (Haudek et al., 2009). Again, this suggests that ROCK function in leukocytes plays an important role in ROCK-dependent responses.

In contrast to the proposed role of ROCK1 in cardiac fibrosis, ROCK2 was shown to be an important player in cardiac hypertrophy. Mice with a cardiomyocyte-specific deletion of ROCK2 display normal cardiac anatomy, function and hemodynamic parameters under basal conditions. Following induction of cardiac hypertrophy induced by angiotensin II infusion or TAC, those mice exhibited substantially less cardiac hypertrophy, intraventricular fibrosis, cardiac apoptosis, and oxidative stress compared to control mice. The reduced hypertrophic phenotype observed in cardiac-specific ROCK2-deficient mice may be due to the upregulation of four-and-a-half LIM-only protein-2 (FHL2). FHL2 was demonstrated to mediate the inhibition of the transcription factor SRF and extracellular signal-regulated mitogen-activated protein kinase (ERK), which are both involved in cardiomyocyte growth. Crossbreeding of haploinsufficient ROCK2^+/–^ and FHL2 KO mice restored the hypertrophic response to angiotensin II (Okamoto et al., 2013).

ROCK2 has recently been implicated in metabolic regulation. After feeding wild type and haploinsufficient ROCK2 mice with a high fat diet for 17 weeks, it was shown that insulin resistance did not develop in the haploinsufficient ROCK2 animals. Moreover, in wild type mice fed with high fat diet, the left ventricular inner diameter in diastole and the myocardial performance index were increased, and left ventricular wall motion dyssynchrony was apparent. None of these changes were present in haploinsufficient ROCK2 mice after high fat diet. This was associated with normalization of insulin signaling and GLUT4 expression in the ROCK2 mouse model (Soliman et al., 2015).

Finally, the complexity of ROCK1 and ROCK2 function in the heart is nicely demonstrated in an analysis of ROCK1 and ROCK2 expression in two models of cardiac pressure overload: pulmonary artery constriction (PAC) and TAC to induce overload in the right (RV) and left ventricle (LV), respectively. PAC induced ROCK2, but not ROCK1 expression in the first days in the RV, which was not evident in the LV. In contrast, TAC induced ROCK1 and ROCK2 expression with a delay of 3 and 7 days, respectively, in the vascular wall and perivascular area. These changes in ROCK expression were accompanied by CD45-positive inflammatory cell infiltration. Transgenic overexpression of a dominant negative Rho-kinase/ROCK2 (DN-RhoK), which inhibits ROCK1 and ROCK2 function, led to significantly fewer changes in pressure-loaded ventricles and improved survival in both PAC and TAC models. Downregulation of the ERK1/2–GATA4 signaling pathway in the DN-RhoK-expressing mice could explain its beneficial effects (Ikeda et al., 2014).

## Function and Regulation of ROCK1 and ROCK2 in Human Cardiovascular Disease

**FIGURE 3:**
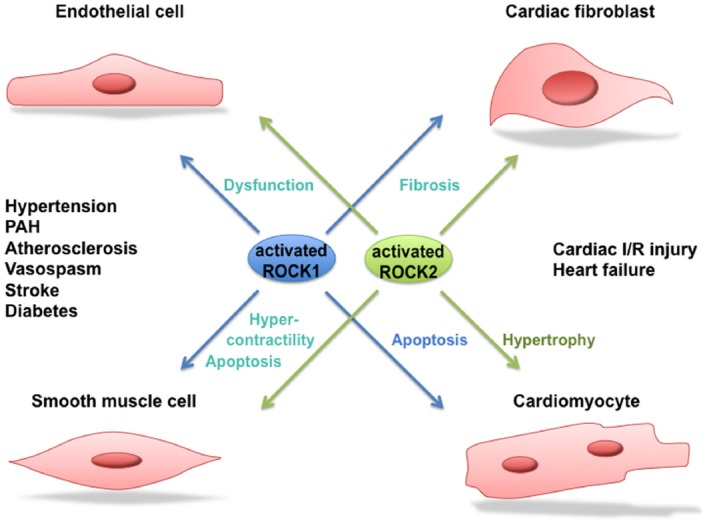
**Role of ROCK1 and ROCK2 in cardiovascular disease.** Activated ROCK1 and ROCK2 play a pivotal role in processes leading to cardiovascular diseases such as hypertension, pulmonary arterial hypertension (PAH), atherosclerosis, vasospastic angina, stroke, diabetes, cardiac ischemia/reperfusion (I/R) injury and heart failure. Where possible, a distinction between the function of ROCK1 and ROCK2 in the different processes is made.

There are several lines of evidence that ROCK activity is increased in patients suffering from hypertension, pulmonary hypertension, stable angina pectoris, vasospastic angina, heart failure, and stroke (Masumoto et al., 2001, 2002; Shimokawa et al., 2002; Kishi et al., 2005; Shibuya et al., 2005; Vicari et al., 2005; Ishikura et al., 2006). An enhanced activity of ROCK signaling was also observed in smokers (Noma et al., 2005). An overview of the involvement of ROCK in human cardiovascular disease can be seen in Figure 3. Where possible, a distinction between ROCK1 and ROCK2 is made.

A number of compounds have been developed to regulate ROCK kinase activity. The most widely used are the non-isoform-selective Y-27632 and fasudil (also known as HA-1077), which act as competitive antagonists for ATP in the kinase domain and do not discriminate between ROCK1 and ROCK2 (Davies et al., 2000). These inhibitors show a relatively high degree of specificity for ROCKs, however, when used at higher concentrations they can also inhibit other kinases, for example protein kinase C-related kinases (PRKs; Davies et al., 2000), citron kinase (Ishizaki et al., 2000), or protein kinase A (Ikenoya et al., 2002). Fasudil is metabolized in the liver to its active metabolite hydroxyfasudil, which has been reported to exert a more specific inhibitory effect on ROCKs than fasudil itself (Rikitake et al., 2005a). In 1995, fasudil was approved in Japan and China for prevention and treatment of cerebral vasospasm following subarachnoid hemorrhage and is currently the only ROCK inhibitor approved for human use. In clinical studies, fasudil shows beneficial effects in patients with PAH, systemic hypertension, vasospastic angina, stroke, and chronic heart failure (Masumoto et al., 2001, 2002; Fukumoto et al., 2005; Kishi et al., 2005; Shibuya et al., 2005). Fasudil was further modified to obtain H-1152P, which is a more specific inhibitor of ROCKs (Sasaki et al., 2002).

Hypertension is one of the most common cardiovascular diseases and is characterized by high arterial pressure due to an increased vascular resistance in the periphery. The reason for this is often elevated vascular contractility and arterial wall remodeling, for example as a consequence of atherosclerosis. Increased activity of the RhoA/ROCK pathway is observed in experimental hypertension models and hypertensive patients (Masumoto et al., 2001; Soga et al., 2011; Smith et al., 2013). For instance, in hypertensive patients treated with the ROCK inhibitor fasudil the peripheral vascular resistance was decreased (Masumoto et al., 2001). The underlying mechanism for the enhanced RhoA/ROCK signaling in hypertension was shown to be a consequence of the upregulated renin-angiotensin-aldosterone system (Seko et al., 2003; Moriki et al., 2004; Wirth et al., 2008; Guilluy et al., 2010), and an increased production of reactive oxygen species (Sun et al., 2008). ROCK inhibitor treatment, including fasudil, in spontaneously hypertensive rats (SHR) decreased the mean arterial blood pressure, but not the systolic blood pressure (Tsounapi et al., 2012). Moreover, ROCKs were also shown to be involved in structural as well as functional alterations of blood vessels in SHR (Mukai et al., 2001). However, ROCK inhibitors do not always lower blood pressure in hypertension models and, as reviewed above, studies utilizing ROCK KO mouse models are quite inconclusive. However, there is strong evidence that ROCK signaling plays an important role in the pathogenesis of hypertension in humans, especially because it was shown that polymorphisms in the ROCK2 gene are associated with a lower risk of developing hypertension (Rankinen et al., 2008).

Treatment of patients suffering from PAH with the ROCK inhibitor fasudil induces acute pulmonary vasodilation, suggesting an involvement of ROCK signaling in PAH (Ishikura et al., 2006). Interestingly, ROCK2 expression is increased in the media of pulmonary arteries and pulmonary arterial smooth muscle cells from patients with idiopathic PAH (Shimizu et al., 2013). This is supported by findings in a rat model for PAH. Long-term treatment with fasudil improved pulmonary hypertension, right ventricular hypertrophy and pulmonary vascular remodeling, as well as survival. This correlated with reduced VSMC proliferation concomitant with increased VSMC apoptosis, lower macrophage infiltration, and attenuated VSMC hypercontractility and endothelial dysfunction (Abe et al., 2004).

Fasudil also appears to improve angina: it increased maximum exercise time and decreased the number of angina attacks per week in patients with stable angina (Shimokawa et al., 2002). Similarly, intracoronary infusion of fasudil attenuated acetylcholine-induced coronary artery spasm and consequently myocardial ischemia in patients with vasospastic angina (Masumoto et al., 2002). These finding are supported by data from porcine models, in which interleukin 1β was chronically applied to coronary arteries from the adventitia to induce inflammatory lesions. Application of fasudil considerably reduced serotonin-induced coronary hyperconstriction. Increased activation of the phosphatase MYPT1, which is normally inhibited by ROCK phosphorylation, is the likely cause of these effects (Katsumata et al., 1997; Shimokawa et al., 1999; Kandabashi et al., 2000).

Another clinical study investigated the effects of exogenous NO on ROCK activity in patients with angina pectoris (Maruhashi et al., 2015). Although exogenous NO is used intensively in the clinic as an anti-anginal agent to dilate the vasculature (Abrams, 2005), its long-term effect is still controversial [ISIS-4 (Fourth International Study of Infarct Survival) Collaborative Group, 1995; Mahmarian et al., 1998; Nakamura et al., 1999]. In this study, ROCK activity in peripheral leukocytes was decreased in patients receiving isosorbide mononitrate as NO donor, while ROCK1 and ROCK2 expression levels were not altered. This indicates that the treatment with exogenous NO could have a beneficial effect in patients with angina pectoris (Maruhashi et al., 2015).

In heart failure patients, administration of fasudil decreased forearm vascular resistance, accompanied by an enhanced vasodilation of the forearm, indicating that ROCK might be involved in the pathogenesis of events leading to heart failure (Kishi et al., 2005). Indeed, ROCK activity is enhanced in patients with acute and chronic heart failure. ROCK activity as measured by the levels of phosphorylated to total MYPT1 in circulating leukocytes (Liu and Liao, 2008) is increased in patients with chronic heart failure and left ventricular remodeling. This enhanced activity is accompanied by systolic dysfunction (Ocaranza et al., 2011). In addition, ROCK activity is increased in congestive heart failure patients and might be associated with mortality (Dong et al., 2012). ROCK is also activated in patients with acute heart failure and seems to decrease during the time course of the disease. However, no significant correlation was found between ROCK activity in heart failure patients and established biomarkers of heart failure, as for instance cardiac troponin I and brain natriuretic peptide (BNP; Do et al., 2013). However, ROCK activity is increased in patients with myocardial infarction, and in combination with high N-terminal pro-B-type natriuretic peptide could be used as a biomarker to identify patients suffering from acute coronary syndrome that might be at high risk (Dong et al., 2013).

Several animal models of heart failure support these findings. Treatment of hypertensive rats with congestive heart failure with the ROCK inhibitor Y-27632 attenuated vascular remodeling and cardiac dysfunction (Kobayashi et al., 2002). Moreover, mice that underwent myocardial infarction operation and were treated with fasudil showed improved left ventricular function. In addition, cardiomyocyte hypertrophy and interstitial fibrosis was decreased, accompanied by decreased expression of inflammatory cytokines (Hattori et al., 2004).

There is increasing evidence that inhibition of ROCK leads to several cardioprotective events in cultured cardiomyocytes from rat or mouse. For example, ROCK inhibition leads to improved cardiac contractility due to diminished phosphorylation of cardiac troponin I/T254, thereby preserving SERCA2a expression (Vlasblom et al., 2009).

In addition, inhibition of ROCK was shown to inhibit cardiomyocyte hypertrophy through attenuated angiotensin II and endothelin-1 signaling (Kuwahara et al., 1999; Yanazume et al., 2002; Hunter et al., 2009; Ye et al., 2009) and apoptosis via activation of PI3K/Akt and ERK/MAPK pathways (Chang et al., 2006; Shi et al., 2010), as well as suppression of the pro-apoptotic Bcl-2 family protein Bax (Del Re et al., 2007). Moreover, inhibition of ROCK reduced cardiac fibrosis via the decreased expression of fibrotic or inflammatory cytokines (Zhang et al., 2006) and greatly reduces vascular resistance by decreasing MLC phosphorylation (Kishi et al., 2005).

Taken together, these findings suggest that ROCK activity in leukocytes may be a new biomarker to predict cardiovascular events, including arterial hypertension, PAH, angina pectoris, vasospastic angina and heart failure. This may reflect a crucial contribution of leukocyte infiltration to the development of cardiovascular diseases. ROCK is also a potentially valuable therapeutic target for cardiovascular disease.

## Conclusions and Future Directions

Since the discovery of ROCKs, a broad range of ROCK substrates involved in diverse cellular responses has been identified. The function of both kinases has long been considered to be similar, however, it has become evident that in addition to common functions, they also have different targets. Moreover, depending on their subcellular localization, activation and other environmental factors, ROCK signaling might lead to different effects on cellular function. In 2008 the first ROCK2-selective inhibitor SLx-2119 was described, which decreases the expression of CTGF in human dermal fibroblasts and thus supports a role for ROCK2 in fibrotic processes (Boerma et al., 2008). This could be a useful inhibitor for dissecting ROCK2 function, but it will need further characterization to determine its specificity. However, more selective inhibitors for ROCK1 versus ROCK2 are needed to allow testing *in vitro* and in clinical studies. Indeed, the usage of selective ROCK inhibitors to treat cardiac diseases could reduce the side effects that are seen with other inhibitors like fasudil. In addition, it will be important to further determine selective functions of ROCK1 and ROCK2, using conditional ROCK1 and ROCK2 depletion in specific tissues or cell types in mice, e.g., in cardiac fibroblasts or cardiomyocytes. Moreover, applying the CRISPR/Cas technology to selectively eliminate each ROCK in human tissue samples would undoubtedly bring new evidence regarding their distinct signaling. Finally, there is still an incomplete picture of how and in which processes ROCKs act as kinases or simply as scaffolding proteins. In that regard, kinase-dead knock-in mice would be a useful tool to study ROCK scaffolding functions. To conclude, there is growing evidence that the ROCK pathway plays an important role in the development of cardiovascular diseases and that inhibition of ROCK activity by selective ROCK inhibitors would be beneficial in treating cardiovascular diseases.

### Conflict of Interest Statement

The authors declare that the research was conducted in the absence of any commercial or financial relationships that could be construed as a potential conflict of interest.
